# The impact of low education and poor health on unemployment varies by work life stage

**DOI:** 10.1007/s00038-017-0972-7

**Published:** 2017-04-18

**Authors:** Sander K. R. van Zon, Sijmen A. Reijneveld, Carlos F. Mendes de Leon, Ute Bültmann

**Affiliations:** 1 Department of Health Sciences, Community and Occupational Medicine, University Medical Center Groningen, University of Groningen, Groningen, The Netherlands; 2 Center for Social Epidemiology and Population Health, University of Michigan School of Public Health, Ann Arbor, MI USA

**Keywords:** Physical health, Mental health, Unemployment, Education, Work life stage, Interaction

## Abstract

**Objectives:**

The aim of this study is to examine associations and interactions of education, and physical and mental health with unemployment in early, mid, and late work life.

**Methods:**

This cross-sectional study uses data from 69,118 respondents from Lifelines. Health status was measured with the RAND-36, education was self-reported, and participants working <12 h per week or indicating to be unemployed were considered unemployed. The relative excess risk due to interaction (RERI) was calculated to measure interaction on the additive scale.

**Results:**

Interactions of low education and poor mental health were found in early [RERI: 2.14; 95% confidence interval (CI): 0.63, 3.65], mid (1.41; 0.61, 2.20) and late (0.63; 0.09, 1.17) work life. Interaction between low education and poor physical health was only found in mid-work life (1.27; 0.61, 1.93).

**Conclusions:**

Low education and poor physical and mental health exacerbate each other’s impact on unemployment varying by work life stage. Policies addressing unemployment may become more effective if they better account for the physical and mental health status of adults in certain stages of their work life.

**Electronic supplementary material:**

The online version of this article (doi:10.1007/s00038-017-0972-7) contains supplementary material, which is available to authorized users.

## Introduction

Employment status is a major social determinant of health and well-being (McKee-Ryan et al. 2005; Bowling 1995). Employment provides financial security, the opportunity to fulfil a social role, and is important for physical and mental health (McKee-Ryan et al. 2005; Bowling 1995). There is considerable evidence that unemployment is associated with lower levels of self-rated physical and mental health (McKee-Ryan et al. 2005; Norström et al. 2014) and reduced survival (Roelfs et al. 2011). In addition to affecting individual health and well-being, unemployment poses a substantial economic burden on society (OECD 2014). A better understanding of the determinants of unemployment among adults could help reduce its burden on individuals and society through better tailored interventions and social policies.

Low education is one of the most important determinants of employment status (Robroek et al. 2013; Schuring et al. 2013; Thielen et al. 2013; Alavinia and Burdorf 2008; Siegrist et al. 2007; Barham et al. 2009; OECD 2010). A recent report from the Organization for Economic Co-operation and Development (OECD) indicated that risks for unemployment across Europe and the US are up to four times higher for lower educated adults compared to their better educated counterparts (OECD 2010). It is more difficult to enter the labour market for both younger and older adults with low education relative to their age peers with higher levels of education (Barham et al. 2009). Staying in the labour market is also more difficult for individuals with lower levels of education. Studies have shown that once in the workforce, low educated individuals are at increased risk for transition from paid employment into unemployment, disability pension, and early retirement (Schuring et al. 2013; Alavinia and Burdorf 2008).

Poor health may result from unemployment, i.e. “health causation”, but may also be an important determinant of employment status, i.e. “health selection” (Burgard and Lin 2013). Across Europe and the US, poor general physical and mental health status, and chronic health conditions such as type 2 diabetes mellitus (T2DM) and cardiovascular disease (CVD), have been associated with unemployment, a decrease in work participation, absenteeism, and early retirement (Robroek et al. 2013; Schuring et al. 2013; Thielen et al. 2013; Alavinia and Burdorf 2008; Siegrist et al. 2007; Kaspersen et al. 2016; Breton et al. 2013; Kouwenhoven-Pasmooij et al. 2016).

In addition, low education and poor health may interact to exacerbate their impact on unemployment beyond the sum of their individual effects (Ahlbom and Alfredsson 2005), although evidence on this issue is scarce. For lower educated people, poor health may pose a particular challenge to enter the labour market, to remain employed, and to re-enter the labour market after unemployment. For them, work activities may be more physically or mentally demanding (Hämmig and Bauer 2013), individual-tailored work-arrangements necessary to gain or keep employment in the context of poor health may be more exceptional (Burgard and Lin 2013), or psychological resources to overcome these challenges may be lacking (Taylor and Seeman 1999; Kristenson et al. 2004). Knowledge about interaction between low education and poor health would offer cues to better tailor interventions and social policies aiming at improvement of the labour market position of vulnerable groups.

The individual and interactive associations of education and poor health with unemployment may also vary importantly by age group. For example, educational attainment levels have been rising steadily across successive birth cohorts in the Netherlands, such that in 2010, 83% of the 25–34 years old had completed at least an upper secondary education compared with 61% of the 55–64 years old (OECD 2012). As a result, people with lower educational attainment have become an increasingly smaller but more vulnerable group for participation in the labour market. In addition, the labour market itself has changed, with increasingly fewer employment opportunities for those at the lower end of the educational spectrum (Barham et al. 2009).

In addition to a cohort effect for education, poor health may show an age effect on employment. That is, poor physical health may be a greater disadvantage at younger ages, as the pool of physically healthy workers is much larger in this age group than in older age groups, where deficiencies in physical health are more common (Niccoli and Partridge 2012; Sacker et al. 2005). In contrast to poor physical health, poor mental health tends to be more common in younger rather than older adult age groups (Chandola et al. 2007; Whiteford et al. 2015). At the same time, poor mental health may be a particularly difficult barrier in the earlier stages of employment, when people have less accumulated work experience to compensate for mental health difficulties in the fulfilment of everyday work requirements (Barham et al. 2009; Eichhorst et al. 2014). In addition, adults with poorer physical or mental health may select themselves into less challenging occupations over time (Burgard and Lin 2013), making poor mental health a smaller threat to their employment than in earlier stages of one’s career. In sum, the combined adverse effects of low education and poor health may be particularly challenging for younger adults seeking or trying to maintain employment, and these effects may interact with one another to a greater degree in these age groups than later in adulthood.

The main aim of this study is to assess the individual and interactive effects of low education and poor physical and mental health with unemployment within specific stages of work life, being early, mid, and late work life. Based on the importance of a high educational level and good health for successful participation in the workforce, we hypothesize that low education and poor health status exacerbate each other’s risk of unemployment, and that this exacerbation is stronger in younger than in older adults.

## Methods

### Study design and sample

The study was conducted using data from the Lifelines Cohort Study (Stolk et al. 2008; Scholtens et al. 2015). Lifelines is a multi-disciplinary prospective population-based cohort study using a unique three-generation design to examine the health and health related-behaviours of 167,729 persons living in the north of The Netherlands. It employs a broad range of investigative procedures in assessing the biomedical, socio-demographic, behavioural, physical and psychological factors which contribute to the health and disease of the general population, with a special focus on multi-morbidity and complex genetics. Participants were recruited between November 2006 and December 2013 through invitations by their general practitioner or family members. In addition, there was an option to self-register. Recruitment and data collection have been described elsewhere (Scholtens et al. 2015). Lifelines was conducted according to the guidelines in the Declaration of Helsinki and all procedures involving human subjects were approved by the Medical Ethics Committee of the University Medical Center Groningen. Written informed consent was obtained from all participants.

The current study uses data from adult participants of working age (≥25–<65 years old) who visited the research centres between November 2006 and March 2013 for the baseline measurements.

### Measures and procedures

**Fig. 1:**
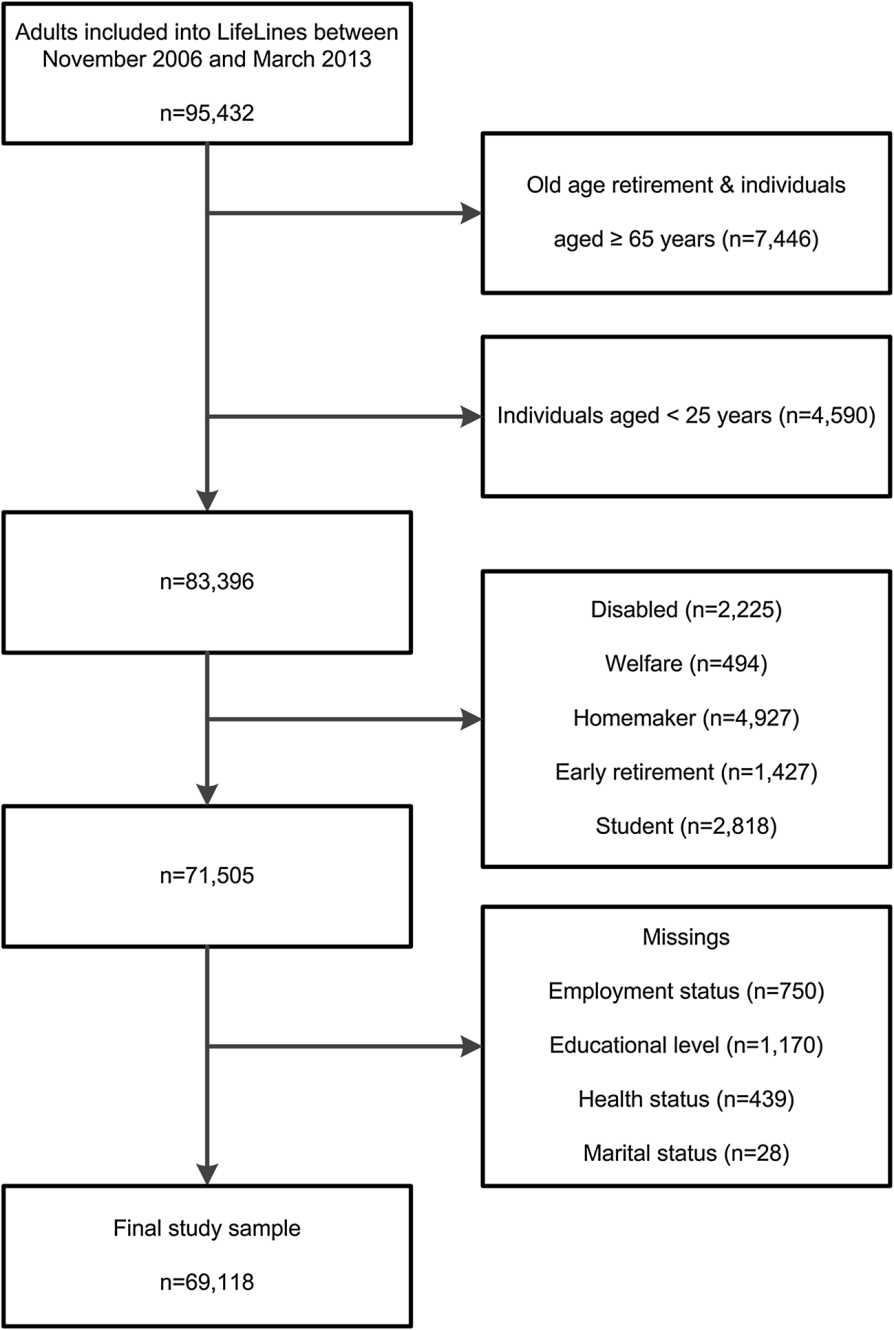
Selection of the analytic study sample (The Netherlands 2006–2013)

Employment status was assessed with the following question: “Which situation applies to you?”: work ≥32 h per week, work ≥20–<32 h per week, work ≥12–<20 h per week, work <12 h per week, unemployed, disabled, on welfare, homemaker, student, early retirement. In line with Statistics Netherlands (Janssen and Dirven 2015), we considered participants working ≥12 h per week being employed and those working <12 h per week and those unemployed as unemployed. Participants indicating to be disabled, on welfare, homemaker, with early retirement, or student were excluded from the analysis. More details on the selection of the analytic study sample are shown in Fig. 1.

Educational level was determined with a single-item question regarding the highest educational level achieved and was categorized into low education (no education, primary education, lower or preparatory vocational education, lower general secondary education), medium education (intermediate vocational education or apprenticeship, higher general senior secondary education or pre-university secondary education), and high education (higher vocational education, university).

Physical and mental health status was measured with the RAND-36 (Hays and Morales 2001). This questionnaire has shown good reliability and validity (Van der Zee et al. 1996). The RAND-36 measures eight health domains with 36 questions. Each domain was scored from 0 to 100 with higher scores indicating better health. The domain scores were standardized by linear *z* score transformation to have a mean of 50 and a standard deviation (SD) of 10 in the US general population (Hays et al. 1998; Ware et al. 1994). A physical component score (PCS) and a mental component score (MCS) were constructed from these eight health domains using recommended scoring algorithms, with all domains contributing to both summary scores (Hays et al. 1998). The PCS primarily reflects measures of physical functioning, pain, and role limitations caused by physical health problems. The MCS primarily reflects measures of emotional well-being and role limitations caused by emotional problems. General health perceptions, energy/fatigue, and social functioning are reflected in both component scores (Hays et al. 1998). Physical and mental health status were dichotomized into poor and good health (PCS and MCS <50 and ≥50, respectively).

Age was calculated based on the date of the first clinical visit. Subsequently, age was divided into three age groups representing early, mid, and late work life. Those aged 25–34 years were considered to be in early work life, those aged 35–49 years in mid-work life, and those aged 50–64 years in late work life.

Marital status was measured with a single question, and dichotomized into living in couple (married/registered partnership, cohabiting) and not living in couple (single, widow/widower, divorced, in a relationship but not cohabiting).

### Statistical analyses

First, we described socio-demographic characteristics for the three age groups representing the different stages of work life.

Second, we assessed the associations of education and health with unemployment using binary logistic regression models. We computed odds ratios (ORs) with 95% confidence intervals (CIs) for each combination of educational level and health status with the category of high education and good health serving as a reference category (Knol and VanderWeele 2012). These and all following analyses were performed separately for physical and mental health, and for the three work life stages, and were adjusted for age, sex, and marital status.

Third, we conducted formal tests of the interaction between education and health with unemployment. Interactions were computed on the additive scale because this is thought to best represent biological interaction (Ahlbom and Alfredsson 2005). In addition, it is generally accepted that assessing interactions on an additive scale is more informative from a public health perspective than interactions on a multiplicative scale (Rothman et al. 2013; Kendler and Gardner 2010; Ahlbom and Alfredsson 2005). To examine interactions of education and health with unemployment, multinomial logistic regression analyses were performed to obtain regression coefficients and the asymptotic covariance matrix. The recommended syntax from Andersson et al. (2005) was used for these analyses. The model was specified as *i* = 1 when low education was present and 0 when high education was present, and *j* = 1 when poor health was present and 0 when good health was present. OR_*ij*_ was the OR in exposure category *i.j*. Three ORs (i.e. OR_11_, OR_10_, and OR_01_), with OR_00_ as the reference category, were estimated. The relative excess risk due to interaction (RERI) was calculated with the formula: RERI = OR_11_–OR_10_–OR_01_ + 1 using the regression coefficients and covariance matrix obtained from the multinomial logistic regression analysis (Andersson et al. 2005). The 95% CI for the RERI was calculated with the delta method using the algorithm by Andersson (Andersson et al. 2005; Hosmer and Lemeshow 1992). Interaction was present when the 95% CI of the RERI did not include 0. The same analyses were performed for medium education as risk factor.

Fourth, as suggested by Knol and VanderWeele (2012) we also assessed the associations of education and unemployment in categories of health (i.e. poor and good health), and the associations of health and unemployment in categories of education (i.e. low, medium, and high education). In contrast to the analysis described in the second step, these analysis show the effect of education on unemployment separately for participants with poor and good health and the effect of health on unemployment separately for participants with low, medium, and high education. These analyses offer additional information on the nature of the interaction effects (Knol and VanderWeele 2012).

In sensitivity analyses, we repeated the analyses using the international definition for unemployment (Janssen and Dirven 2015), with only considering participants indicating to be unemployed as unemployed.

## Results

### Baseline characteristics

A total of 69,118 participants were included in the study, of whom 19.5% were in their early work life, 57.7% in mid-work life and 22.8% in late work life (Table 1). The prevalence of individuals with low education was almost three times higher for those in late work life than in early work life (38.0 vs. 13.0%). The prevalence of unemployment was highest in late work life (11.6%) and lowest in early work life (5.9%). The prevalence of people with poor physical health increased stepwise by work life stage from 20.7% in early to 30.4% in late work life. The opposite was observed for mental health (27.1–20.2%).

**Table 1:** Baseline characteristics of the study population by work life stage (The Netherlands 2006–2013)

	Work life stage		
	Early (25–34)	Mid (35–49)	Late (50–64)
	(*n* = 13,479)	(*n* = 39,879)	(*n* = 15,760)
Age [mean (SD)]	29.9 (2.8)	42.8 (4.2)	54.6 (4.1)
Female gender (%)	57.1	56.4	53.2
Living in couple (%)	74.7	86.0	87.8
Educational level (%)			
High	44.5	30.5	28.3
Medium	42.5	43.6	33.7
Low	13.0	25.8	38.0
Unemployed (%)			
Dutch definition	5.9	7.1	11.6
International definition	3.4	2.5	3.6
PCS [mean (SD)]	52.7 (6.2)	51.8 (6.7)	50.9 (6.9)
Poor PCS (%)	20.7	25.4	30.4
MCS [mean (SD)]	51.8 (8.2)	52.5 (8.2)	53.6 (7.7)
Poor MCS (%)	27.1	24.4	20.2

*SD* standard deviation, *PCS* physical component score, *MCS* mental component score

### Associations and interactions of education and health with unemployment

As expected, risk of unemployment tended to increase with increasingly lower levels of education within each category of health, and at each stage of work life (Table 2). This pattern held for both physical health and mental health. For physical health, the highest odds of unemployment were observed for those with low education and poor health in early work (OR: 4.72; 95% CI 3.52, 6.33) and mid-work life (OR: 5.00; 95% CI 4.31, 5.81), respectively. However, the absolute risk of unemployment for those with low education and poor physical health was highest in late work life (20.2%), followed by early work life (14.9%) and mid-work life (14.6%). Interaction between low education and poor physical health was observed in mid-work life (RERI: 1.27; 95% CI 0.61, 1.93), and extended to medium education in early (RERI: 0.69; 95% CI 0.10, 1.27) and mid (RERI: 0.89; 95% CI: 0.45, 1.33) work life.

**Table 2:** Associations and interactions of education, and physical and mental health on unemployment, stratified by work life stage: odds ratios for unemployment per category of health and work life stage and associated RERIs’ (The Netherlands 2006–2013)

	Physical health					Mental health			
	*n* unemployed/*n* total (% unemployed)		OR (95% CI)	RERI (95% CI)		*n* unemployed/*n* total (% unemployed)		OR (95% CI)	RERI (95% CI)
Early work life									
Good health	557/10,693	(5.2)				458/9824	(4.7)		
High education	190/5016	(3.8)	1 (Ref)			151/4496	(3.4)	1 (Ref)	
Medium education	242/4406	(5.5)	1.57 (1.29, 1.91)			204/4134	(4.9)	1.58 (1.27, 1.96)	
Low education	125/1271	(9.8)	3.24 (2.55, 4.12)			103/1194	(8.6)	3.18 (2.44, 4.14)	
Poor health	234/2786	(8.4)				333/3655	(9.1)		
High education	46/984	(4.7)	1.13 (0.81, 1.58)			85/1504	(5.7)	1.52 (1.16, 2.00)	
Medium education	116/1320	(8.8)	2.39 (1.87, 3.04)	0.69 (0.10, 1.27)^a^		154/1592	(9.7)	2.88 (2.28, 3.64)	0.78 (0.15, 1.41)^c^
Low education	72/482	(14.9)	4.72 (3.52, 6.33)	1.36 (−0.04, 2.77)^b^		94/559	(16.8)	5.81 (4.40, 7.68)	2.14 (0.63, 3.65)^d^
Mid-work life									
Good health	1774/29,763	(6.0)				1818/30,151	(6.0)		
High education	325/9781	(3.3)	1 (Ref)			268/9311	(2.9)	1 (Ref)	
Medium education	759/12,950	(5.9)	1.72 (1.50, 1.97)			826/13,266	(6.2)	2.12 (1.84, 2.44)	
Low education	690/7032	(9.8)	3.34 (2.91, 3.84)			724/7574	(9.6)	3.77 (3.26, 4.36)	
Poor health	1065/10,116	(10.5)				1021/9728	(10.5)		
High education	122/2400	(5.1)	1.38 (1.12, 1.71)			179/2870	(6.2)	1.95 (1.61, 2.38)	
Medium education	464/4440	(10.5)	2.99 (2.58, 3.46)		0.89 (0.45, 1.33)^a^	397/4124	(9.6)	3.04 (2.58, 3.57)	−0.03 (−0.51, 0.46)^c^
Low education	479/3276	(14.6)	5.00 (4.31, 5.81)		1.27 (0.61, 1.93)^b^	445/2734	(16.3)	6.13 (5.22, 7.20)	1.41 (0.61, 2.20)^d^
Late work life									
Good health	1107/10,965	(10.1)				1391/12,571	(11.1)		
High education	195/3371	(5.8)	1 (Ref)			235/3587	(6.6)	1 (Ref)	
Medium education	309/3671	(8.4)	1.53 (1.26, 1.84)			380/4240	(9.0)	1.43 (1.20, 1.70)	
Low education	603/3923	(15.4)	2.54 (2.13, 3.01)			776/4744	(16.4)	2.42 (2.07, 2.83)	
Poor health	714/4795	(14.9)				430/3189	(13.5)		
High education	103/1086	(9.5)	1.53 (1.19, 1.97)			63/870	(7.2)	1.10 (0.82, 1.47)	
Medium education	194/1644	(11.8)	2.02 (1.63, 2.50)		−0.04 (−0.55, 0.47)^a^	123/1075	(11.4)	1.83 (1.45, 2.32)	0.31 (−0.20, 0.82)^c^
Low education	417/2065	(20.2)	3.50 (2.72, 3.42)		0.44 (−0.10, 0.98)^b^	244/1244	(19.6)	3.14 (2.58, 3.84)	0.63 (0.09, 1.17)^d^

ORs and RERIs are adjusted for age, gender and marital status

*OR* odds ratio, *RERI* relative excess risk due to interaction

^a^RERI of unemployment for medium education and poor physical health

^b^RERI of unemployment for low education and poor physical health

^c^RERI of unemployment for medium education and poor mental health

^d^RERI of unemployment for low education and poor mental health

For mental health, the highest odds of unemployment were observed for those with low education and poor health in early (OR: 5.81; 95% CI 4.40, 7.68) and mid-work life (OR: 6.13; 95% CI 5.22, 7.20). However, as was found for physical health, the absolute risk of unemployment for those with low education and poor mental health was highest in late work life (19.6%), followed by early work life (16.8%) and mid-work life (16.3%). Interaction between low education and poor mental health was observed across all stages of the work life, with the interaction effect decreasing from a RERI of 2.14 (95% CI 0.63, 3.65) in early work life to 1.41 (95% CI 0.61, 2.20) and 0.63 (95% CI 0.09, 1.17) in mid- and late-work life, respectively.

### Stratified analysis of unemployment within categories of health and education

In analyses stratified by good versus poor physical health, increasingly lower levels of education were consistently associated with higher odds of unemployment within each category of health (Table 3). The magnitude of these associations was stronger in early and mid-work life relative to the late work life. A very similar pattern was found for mental health.

**Table 3:** Associations of education and unemployment within categories of physical and mental health, stratified by work life stage (The Netherlands 2006–2013)

	Physical health	Mental health
	OR (95% CI)	OR (95% CI)
Early work life		
Good health		
High education	1 (Ref)	1 (Ref)
Medium education	1.58 (1.30, 1.92)	1.60 (1.29, 1.99)
Low education	3.28 (2.58, 4.17)	3.31 (2.54, 4.32)
Poor health		
High education	1 (Ref)	1 (Ref)
Medium education	2.09 (1.47, 2.98)	1.86 (1.41, 2.45)
Low education	4.08 (2.75, 6.06)	3.64 (2.65, 4.99)
Mid-work life		
Good health		
High education	1 (Ref)	1 (Ref)
Medium education	1.71 (1.49, 1.95)	2.10 (1.82, 2.42)
Low education	3.35 (2.92, 3.85)	3.81 (3.29, 4.41)
Poor health		
High education	1 (Ref)	1 (Ref)
Medium education	2.16 (1.76, 2.66)	1.56 (1.30, 1.88)
Low education	3.56 (2.89, 4.39)	3.05 (2.53, 3.66)
Late work life		
Good health		
High education	1 (Ref)	1 (Ref)
Medium education	1.53 (1.27, 1.85)	1.44 (1.21, 1.72)
Low education	2.51 (2.11, 2.99)	2.38 (2.03, 2.78)
Poor health		
High education	1 (Ref)	1 (Ref)
Medium education	1.31 (1.01, 1.70)	1.64 (1.19, 2.25)
Low education	2.30 (1.82, 2.91)	2.96 (2.20, 3.97)

ORs are adjusted for age, gender and marital status

Some categories in this Table are similar to those in Table 2 but ORs may slightly differ because these are within category analyses (i.e. different size of the sample being analyzed and therefore a slightly different correction for age, gender, and marital status)

*OR* odds ratio

In analyses stratified by education, poor physical health tended to be associated with higher odds of unemployment across stages of the work life, although these associations were less robust in early work life (Table 4). Again, we found a very similar pattern for mental health. Associations of poor mental health with unemployment reached statistical significance at all levels of education at each stage of work life, except for participants in late work life with poor mental health but a high education. In early and mid-work life, the magnitude of associations between poor health and unemployment by level of education was somewhat greater for mental health than physical health. If anything, this pattern was reversed in late work life.

**Table 4:** Associations of physical, and mental, health and unemployment within categories of education, stratified by work life stage (The Netherlands 2006–2013)

	Physical health	Mental health
	OR (95% CI)	OR (95% CI)
Early work life		
High education		
Good health	1 (Ref)	1 (Ref)
Poor health	1.21 (0.87, 1.68)	1.59 (1.21, 2.10)
Medium education		
Good health	1 (Ref)	1 (Ref)
Poor health	1.50 (1.19, 1.90)	1.80 (1.44, 2.24)
Low education		
Good health	1 (Ref)	1 (Ref)
Poor health	1.42 (1.03, 1.95)	1.80 (1.32, 2.45)
Mid-work life		
High education		
Good health	1 (Ref)	1 (Ref)
Poor health	1.40 (1.13, 1.74)	2.03 (1.66, 2.47)
Medium education		
Good health	1 (Ref)	1 (Ref)
Poor health	1.74 (1.54, 1.97)	1.43 (1.26, 1.63)
Low education		
Good health	1 (Ref)	1 (Ref)
Poor health	1.49 (1.31, 1.69)	1.60 (1.40, 1.82)
Late work life		
High education		
Good health	1 (Ref)	1 (Ref)
Poor health	1.58 (1.23, 2.04)	1.15 (0.85, 1.55)
Medium education		
Good health	1 (Ref)	1 (Ref)
Poor health	1.34 (1.11, 1.63)	1.29 (1.03, 1.61)
Low education		
Good health	1 (Ref)	1 (Ref)
Poor health	1.38 (1.20, 1.60)	1.28 (1.08, 1.51)

ORs are adjusted for age, gender and marital status

Some categories in this Table are similar to those in Table 2 but ORs may slightly differ because these are within category analyses (i.e. different size of the sample being analyzed and therefore a slightly different correction for age, gender, and marital status)

*OR* odds ratio

### Sensitivity analysis

For the sensitivity analysis, we adopted the international definition of unemployment, which is less strict than our original definition. As a result, unemployment rates were substantially lower than those in the main analysis, ranging from about 1–10%, instead of from 3–20% (Online Resource 1). In addition, ORs for unemployment were generally lower for both physical and mental health across all categories of education. However, the general pattern of interaction effects was similar to the one observed in the main analysis. Several of these interaction effects became larger and statistically more robust in these analyses, especially for poor physical and mental health in early and mid-work life.

## Discussion

Our study shows that low education, as well as poor physical and mental health are independently associated with unemployment across different stages of work life. In addition, we found some evidence that on an additive scale, low education and poor health exacerbate each other’s negative impact on unemployment. The interaction effects were most consistent for adults in early and mid-work life, even if the findings in mid-work life were statistically more robust, likely due to differences in sample size. Poor health also appeared to exacerbate the effects of low education on unemployment in late work life, especially poor mental health.

We found an interaction between low education and poor physical health on unemployment for participants in mid-work life. That may be explained in several ways. First, it may be more difficult to enter, stay in, or re-enter the labour force for lower educated individuals when faced with poor physical health because they may have less job control or because work activities may be more physically demanding (Burgard and Lin 2013; Hämmig and Bauer 2013). Second, individual-tailored work-arrangements necessary to gain or keep employment in the context of poor health may be more exceptional (Burgard and Lin 2013). Third, psychological resources to overcome the challenge of finding or keeping paid employment in the context of poor health may be lacking (Taylor and Seeman 1999; Kristenson et al. 2004). These explanations take place at the individual and labour market level and are likely to both contribute to the interaction we found, with the first explanation as the most likely driver.

The absence of an interaction between low education and poor physical health in early and late work life may be explained by an age effect, at least partly. Normally, the pool of healthy workers is much larger in mid than in late work life (Niccoli and Partridge 2012). Selection into unemployment, based on health, may thus be more likely for those in their mid-work life, especially when they have a low education. Following this line of reasoning, interaction would also be expected in participants in early work life but more time might be needed for this selection process to happen. Sensitivity analyses did show interaction between low education and poor physical health in early work life however. This might be explained by the less strict definition for being employed, which may have resulted in catching an even more vulnerable group of individuals in the unemployment group.

The interaction between low education and poor mental health on unemployment was more stable across the work life stages than regarding physical health. We found an interaction for participants in early, mid, and late work life, with the interaction effect decreasing with work life stage. Similar explanations at the individual and labor market level as previously discussed for physical health may explain these interactions (Burgard and Lin 2013; Hämmig and Bauer 2013; Taylor and Seeman 1999; Kristenson et al. 2004). The notion that good mental health is necessary for all job types across the work life course may explain the finding of interactions between low education and poor mental health across all work life stages. The decrease of the interaction effect by work life stage may be due to self-selection into less challenging occupations over time, making poor mental health a smaller threat for unemployment in late than in earlier work life. The decrease in interaction effect by work life stage may also be explained by our exclusion from the study sample of those reporting to be disabled, on welfare or with early retirement. These exit routes from the labour market may be used more often in case of poor health, in particular by low educated people. This health selection effect may lead to an underestimation of the relationship between poor health and unemployment, and may thus also at least partly explain the decreasing interaction effect by work life stage.

We showed that low education, and poor physical and mental health were separately associated with unemployment. This is in line with previous studies that firmly established that low education, and poor physical and mental health are major determinants of employment status (e.g. unemployment, early retirement) (Robroek et al. 2013; Schuring et al. 2013; Thielen et al. 2013; Alavinia and Burdorf 2008; Siegrist et al. 2007; Kaspersen et al. 2016; Breton et al. 2013; Kouwenhoven-Pasmooij et al. 2016). The relationship between education and employment status is typically straightforward, i.e. low education is related to an unfavourable employment status. Reverse causation thus seems unlikely to explain our findings. The relationship between health and employment status is far more complicated (Burgard and Lin 2013). On the one hand, exposures and experiences associated with work may affect health status, i.e. “health causation”, on the other hand, health may affect employment status, i.e. “health selection”. Moreover, the direction of the relationship may change during the life course. Research with longitudinal data is therefore needed to confirm our results and to rule out reverse causation.

We further found that the prevalence of poor mental health decreased by age group while the prevalence of poor physical health increased. This is in line with previous research showing that general mental health improves, and general physical health deteriorates, from young to middle and early older ages (Myint and Welch 2012). This trend for general mental health status differs from the general trend for severe mental health problems like depression (Statistics Netherlands 2016), implying that findings could be different if addressing such a specific diagnosis instead of general mental health.

This study has some major strengths worth mentioning. First, the large sample size allowed precise estimates of the associations and interactions of education, and physical and mental health with unemployment. Second, we examined associations and interactions across work life stages. We thereby showed that the importance of educational level and health status, and their combination, may differ for early, mid, and late work life. Third, we investigated the associations and interactions of education and health on unemployment from the Dutch perspective but performed additional analysis to allow for an international comparison, thereby also showing that the definition for unemployment may have some influence on study findings.

This study also has some limitations. First, as in any cross-sectional study we could not establish causation, showing a need to confirm causality with longitudinal data. Second, we had no information on current or previous job type and job characteristics. Information about job demands and flexibility in work arrangements could help to explain the excess risk for unemployment in those with low education and poor physical or mental health.

Our findings imply that social policies addressing unemployment may be more effective if they include the mental health status of lower educated individuals across all stages of the work life more strongly to provide them with better opportunities for, or stay in, paid employment. Regarding physical health, social policies should pay particular attention to individuals in early and mid-work life. In addition, our study confirms the need for social policies aiming to guide adolescents to a sufficient educational level, and to particularly address those in or at risk for poor health, as educational level is again shown to be an important determinant of employment status. Future studies should verify our findings with longitudinal data, preferably from a life course perspective taking the accumulation of health risks and advantages into account (Amick et al. 2016). These studies could also take current and previous work characteristics into account. Period effects due to current changes in the labour market such as less permanent or long-term contracts and more flexible dismissal schemes also deserve attention (OECD 2010).

We conclude that low education, and poor physical and mental health are independently associated with unemployment. More importantly, low education and poor mental health may exacerbate each other’s impact on unemployment across all work life stages whereas low education and poor physical health may exacerbate each other’s impact on unemployment in mid-work life, with a possible extension to early work life. Social policies addressing unemployment may become more effective if they better account for the physical and mental health status of adults in specific stages of their work life.

## Electronic supplementary material

Below is the link to the electronic supplementary material.

Supplementary material 1 (PDF 117 kb)
