# Supplementary material for: The impact of low education and poor health on unemployment varies by work life stage
Source: Int J Public Health. 2017 Apr 18;62(9):997–1006. doi: 10.1007/s00038-017-0972-7 (PMC5668328; doi:10.1007/s00038-017-0972-7)
Supplement: Supplementary file 1 — Supplementary material 1 (PDF 117 kb) [file 38_2017_972_MOESM1_ESM.pdf]

The impact of low education and poor health on unemployment varies by work life stage

International Journal of Public Health

Sander K.R. van Zon[1]*, Sijmen A. Reijneveld[1], Carlos F. Mendes de Leon[2], Ute Bültmann[1]

1. Department of Health Sciences, Community & Occupational Medicine, University Medical Center Groningen, University of Groningen, Groningen, The Netherlands

2. Center for Social Epidemiology and Population Health, University of Michigan School of Public Health, Ann Arbor, Michigan, United States of America

* Corresponding author: Department of Health Sciences, Community & Occupational Medicine, University Medical Center Groningen, Groningen, the Netherlands. E-mail: s.k.r.van.zon@umcg.nl, telephone +31 50-3616676, fax +31 50-3636251.

Supplementary Table 1. Associations and interactions of education, and physical and mental health on unemployment using the international definition for unemployment (The Netherlands, 2006-2013)

	**Physical health**				**Mental health**			
	**n unemployed / n total (% unemployed)**		**OR (95% CI)**	**RERI (95% CI)**	**n unemployed / n total (% unemployed)**		**OR (95% CI)**	**RERI (95% CI)**
**Early work life**								
**Good health**	**316/10,693**	**(2.9)**			**249/9,824**	**(2.5)**		
High education	139/5,016	(2.8)	1 (Ref)		108/4,496	(2.4)	1 (Ref)	
Medium education	123/4,406	(2.8)	1.06 (0.82, 1.35)		89/4,134	(2.2)	0.93 (0.70, 1.23)	
Low education	59/1,271	(4.6)	1.78 (1.30, 2.43)		52/1,194	(4.4)	1.92 (1.36, 2.70)	
**Poor health**	**137/2,786**	**(4.9)**			**209/3,655**	**(5.7)**		
High education	31/984	(3.2)	1.11 (0.75, 1.66)		62/1,504	(4.1)	1.57 (1.14, 2.17)	
Medium education	57/1,320	(4.3)	1.60 (1.17, 2.20)	0.43 (-0.19, 1.06)[a]	91/1,592	(5.7)	2.33 (1.75, 3.10)	0.83 (0.17, 1.49)[c]
Low education	49/482	(10.2)	3.99 (2.83, 5.63)	2.11 (0.75, 3.48)[b]	56/559	(10.0)	4.18 (2.98, 5.87)	1.73 (0.36, 3.09)[d]
**Mid work life**								
**Good health**	**589/29,763**	**(2.0)**			**541/30,151**	**(1.8)**		
High education	138/9,781	(1.4)	1 (Ref)		104/9,311	(1.1)	1 (Ref)	
Medium education	258/12,950	(2.0)	1.42 (1.15, 1.75)		237/13,266	(1.8)	1.61 (1.28, 2.04)	
Low education	193/7,032	(2.7)	1.98 (1.59, 2.48)		200/7,574	(2.6)	2.42 (1.90, 3.07)	
**Poor health**	**414/6,116**	**(6.8)**			**462/9,728**	**(4.7)**		
High education	52/2,400	(2.2)	1.50 (1.09, 2.08)		86/2,870	(3.0)	2.37 (1.78, 3.18)	
Medium education	162/4,440	(3.6)	2.55 (2.02, 3.22)	0.62 (0.005, 1.24)[a]	183/4,124	(4.4)	3.51 (2.74, 4.49)	0.53 (-0.24, 1.31)[c]
Low education	200/3,276	(6.1)	4.49 (3.59, 5.60)	2.03 (1.20, 2.87)[b]	193/2,734	(7.1)	5.80 (4.54, 7.40)	2.04 (0.94, 3.14)[d]
**Late work life**								
**Good health**	**338/10,965**	**(3.1)**			**400/12,571**	**(3.2)**		
High education	74/3,371	(2.2)	1 (Ref)		79/3,587	(2.2)	1 (Ref)	
Medium education	110/3,671	(3.0)	1.46 (1.08, 1.97)		129/4,240	(3.0)	1.49 (1.12, 1.98)	
Low education	154/3,923	(3.9)	1.82 (1.37, 2.41)		192/4,744	(4.0)	1.86 (1.42, 2.42)	
**Poor health**	**225/4,795**	**(4.7)**			**163/3,189**	**(5.1)**		
High education	34/1,086	(3.1)	1.47 (0.97, 2.22)		29/870	(3.3)	1.48 (0.96, 2.29)	
Medium education	69/1,644	(4.2)	2.07 (1.48, 2.90)	0.14 (-0.67, 0.96)a	50/1,075	(4.7)	2.16 (1.50, 3.12)	0.21 (-0.70, 1.13)[c]
Low education	122/2,065	(5.9)	2.78 (2.07, 3.75)	0.50 (-0.28, 1.28)b	84/1,244	(6.8)	3.06 (2.22, 4.20)	0.69 (-0.22, 1.61)[d]

Abbreviations: OR: odds ratio; RERI: relative excess risk due to interaction

ORs and RERIs are adjusted for age, gender and marital status

[a] RERI of unemployment for medium education and poor physical health; [b] RERI of unemployment for low education and poor physical health; [c] RERI of unemployment for medium education and poor mental health; [d] RERI of unemployment for low education and poor mental health
